# Deep Reactive Ion Etching of Z-Cut Alpha Quartz for MEMS Resonant Devices Fabrication

**DOI:** 10.3390/mi11080724

**Published:** 2020-07-26

**Authors:** Bo Li, Cun Li, Yulong Zhao, Chao Han, Quanwei Zhang

**Affiliations:** State Key Laboratory for Manufacturing System Engineering, Xi’an Jiaotong University, Xi’an 710049, China; li.bo.123.666@stu.xjtu.edu.cn (B.L.); zhaoyulong@mail.xjtu.edu.cn (Y.Z.); hc2113@stu.xjtu.edu.cn (C.H.); z553607972@stu.xjtu.edu.cn (Q.Z.)

**Keywords:** quartz deep etching, machining accuracy, Cr mask, vertical sidewall, smooth surface

## Abstract

Quartz is widely used in microelectromechanical systems (MEMS). Especially, MEMS quartz resonators are applied to sensors and serve as sensitive elements. The capability of deep etching is a limitation for the application. Presented in this paper is a deep and high accuracy reactive ion etching method applied to a quartz resonator etching process with a Cr mask. In order to enhance the capability of deep etching and machining accuracy, three kinds of etching gas (C_4_F_8_/Ar, SF_6_/Ar and SF_6_/C4F_8_/Ar), bias power, inductively coupled plasma (ICP) power and chamber pressure were studied in an industrial reactive ion etching machine (GDE C200). Results indicated that the SF_6_/C_4_F_8_/Ar chemistry gas is the suitable and optimal choice. Experiment results indicate that Cr (chromium) mask can obtain a higher selectivity than aluminum and titanium mask. A “sandwich” structure composed of Al layer-Cr layer-Al layer-Cr layer was proposed. The Al (aluminum) film can play the role of releasing stress and protecting gold electrodes, which can enhance the thickness of metal mask. An optimized process using SF_6_/C_4_F_8_/Ar plasmas showed the quartz etching rate of 450 nm/min. Meanwhile, a microchannel with a depth of 75.4 µm is fabricated, and a nearly vertical sidewall profile, smooth surface is achieved.

## 1. Introduction

Quartz has been widely used as a resonator material in micro-sensors [1,2,3] due to its excellent material properties such as its high quality factor [4,5,6,7,8,9,10], high frequency stability and inherent piezoelectric characteristics. A major obstacle limiting the wide use of quartz is that there exists a limited variety of suitable processing methods for producing structures with desired shapes. In order to solve this problem, a conventional method known as wet chemical etching has been studied by several researchers [1,11,12,13], which is mainly performed in aqueous hydrofluoric (HF), and often in combination with ammonium fluoride (NH_4_F) as a buffer. However, the high degree of anisotropy, the trigonal symmetry of the quartz crystal and large etch rate hinder its study and use. Recently, a few micromachining processes such as laser beam machining (LBM) [14], abrasive jet machining (AJM) [15,16] and electrochemical discharge machining (ECDM) [17,18,19,20,21] have been studied for realizing micro-structures on quartz substrate. Though the etching rate is overtly improved with these technologies, it is not suitable for manufacturing complex structures and mass production. Moreover, the laser energy results in a quartz crystal denaturation at the edge of the resonant beam in LBM technology; the roughness and flatness of the etched bottom and sidewall cannot achieve the accuracy requirements of the resonator in LBM and ECDM technology, which induces an undesirable effect on the performance of quartz resonators. Moreover, the machining accuracy is too low to manufacture much smaller micro-resonators. Therefore, a deep dry etching method with inductively coupled plasma has been investigated to obtain an excepted profile [4,5,9,22,23,24,25,26,27,28,29,30,31,32,33,34,35,36].

Several typical masks, such as metal masks, photoresist masks and metallic compound masks have been discussed so far [24,28,34,35,36,37,38,39,40,41,42,43]. It is known that the deep and high etching rate of glasses is not feasible with standard photoresist masks due to low selectivity and mask profile degradation during the prolonged process [37,38]. Therefore, other masks have drawn much attention in recent years. A thin sputtered Al_2_O_3_ (aluminum oxide) mask was studied to achieve high selectivity to SiO_2_ (silicon dioxide) when the gas composition of the C_4_F_8_/SF_6_ mixture, pressure, and bias power was properly tuned [28,38]. However, the original thickness of Al_2_O_3_ was 100 nm with a 20 min deposition process. It was therefore infeasible to acquire a deep etching depth due to the disadvantage of slow sputtering and deposition rate. Moreover, a novel process was designed by periodically oxidizing the surface of an Al mask in oxygen plasma to obtain an Al2O3 mask during the breaks of the SiO_2_ etching process [29,44]. Thus, 5 μm thick silica waveguides were achieved with vertical and smooth profiles using an initial Al mask of only 100 nm. However, the extra oxidizing step resulted in the etching duration increasing and the throughput suffered considerably. In order to achieve a deep etching of SiO_2_, the trade-off between the selectivity and thickness of the mask should be paid attention to.

Owing to the high deposition rate and acceptable selectivity, Cr and Ni were selected as a hard mask for the deep dry etching of SiO_2_. A 27 μm-deep trench has been achieved with a 15 μm Ni hard mask [24], using SF_6_ and SF_6_ /Ar plasmas in an inductively coupled plasma (ICP) reactor. However, the angle and roughness of sidewalls were unsatisfactory, which is an obstacle to using this approach to manufacture resonators. Under optimized etching conditions, deep trench etching of borosilicate glass to a 32 μm depth using an electroplated Cr mask created by wet etching was reported [24]. Nevertheless, the etching depth was less than that required for the quartz resonator. Meanwhile, some investigators have explored the AlN (Aluminum nitride) hard mask to receive a higher selectivity possibility [22,28,34,38]. However, most of the studies concentrate on only one aspect to obtain high selectivity but neglect others. Consequently, more research on improving etching depth and gaining high quality surface profiles should be focused on.

In light of the above research studies, it should be noted that the presented results of SiO_2_ etching process development were performed with amorphous silicon oxide, such as undoped silicon oxide glass (USG), silica, fused quartz and quartz glass. In reality, the material of an electromechanical resonator is the single crystal quartz [13,45,46,47], which is selected due to its piezoelectric characteristic with low-loss elastic and electrical characteristics. Although the chemical composition of the above-mentioned materials is the same, and the deep dry-etching distinctions of them are in existence, more attention should be paid to the deep quartz etch. However, information and investigations on deep dry etching on single crystal quartz substrate for quartz resonators fabrication are scarce so far. Owing to the anisotropic property of alpha quartz, the basic plasma etching process parameters of single crystal quartz are more complex in comparison with those of amorphous silicon oxide. In this case, a systematic research on the deep dry etching of anisotropic single crystal quartz is required, especially the Z-cut alpha quartz. Thus, in this work, we investigate the possibilities for the deep dry etching of the Z-cut alpha quartz crystal with metal masks. The preparation of the thick Cr mask is investigated and a “sandwich” structure is proposed to reduce the residual stress. Additionally, in order to obtain a smooth etched surface and vertical sidewall, the etching process parameters are optimized and tuned; a MEMS quartz resonant sensor is fabricated through the proposed etching technology.

## 2. Experiment

The Z-cut alpha quartz etching process was performed in a deep reactive ion etching (DRIE) system (GDE C200, China, Beijing), which has two high-density 13.56 MHz plasma sources (inductively coupled plasma (ICP) and capacitive coupled plasma (CCP), designed to etch materials which are infeasible to etch using conventional reactive ion etching (RIE) or ICP sources, such as silicon oxide, silicon nitride, aluminum oxide. The ICP source was a planar electrode on the top of the reaction chamber with a spiral coil. The ICP energy was supplied by electromagnetic inductions with a frequency of 13.56 MHz. The CCP source had the same frequency and was placed at the bottom of the reaction chamber. The cooled chuck with electrostatic clamping and helium backside cooling was served as a stage in which the target wafer was kept. The internal diameter of the reactor was 500 mm and the distance from the substrate chuck to the top of the reaction chamber was 150 mm. In this system the ICP produced the plasma of ions, while the CCP was applied to accelerate the ions onto the cathode. In order to guarantee the stability of the etching process, a chiller was employed to maintain the chuck at pre-set temperature.

In order to enable a high quality of the deposited Cr layer, etching targets, and z-cut alpha quartz wafers were subjected to mechanical cleaning through a mixture solution of concentrated sulfuric acid and hydrogen peroxide at 80 °C. Three types of metal hard masks were evaluated in our research. The metal masks were deposited by magnetron sputtering in a multi-cathode sputter deposition platform system (DENTON, DISCOVERY635, Denton, TX, USA). Before the metal layer was created, a photoresist (AZ4620, RDMICRO, SuZhou, China) with a thickness of about 13 μm was spin-coated on the quartz wafer and patterned by photolithography. Then, a subsequent lift-off was applied to stripping the photoresist. 

The length, width and thickness of the quartz wafer (LINDE, Xi’an, China) was 34.5 mm, 29.5 mm and 100 µm, respectively. The diameter of the wafer chuck was 4 inches. Thus, the quartz wafer was fixed on a silicon wafer with perfluoropolyether during the etching process. The wafer chuck temperature was 90 °C. Three etching gas mixtures (C_4_F_8_/Ar, SF_6_/Ar and C_4_F_8_/SF_6_/Ar), bias power, ICP power and chamber pressure were studied in our experiment. Prior to etching, residual debris and organic contaminants were removed from the surface of the wafers with dry nitrogen gas and oxygen plasma, respectively. 

## 3. Results 

### 3.1. Effect of Etching Gas

Three gas mixtures (C_4_F_8_/Ar, SF_6_/Ar and SF_6_/SF_4_/Ar) were investigated with a Cr mask in this paper. The initial set of etching parameters: coil (ICP) power 1600 W, platen (bias) power 200 W, pressure 5 mT, and total gas flow rate fixed at 110 sccm. All gas percentages are in volume. The main challenge of deep quartz etching is in the production of high-energy ions to break the strong Si–O bonds while maintaining acceptable selectivity towards the mask material and vertical profile. Ar gas served as a bombardment component, it enhanced the sputtering component of the etching which led to an increased quartz etching rate. However, the etching selectivity was found to be slightly reduced, possibly because of the increased bombardment rate of the Cr mask at the same time.

#### 3.1.1. Mixture of C_4_F_8_/Ar and SF_6_/Ar 

**Figure 1:**
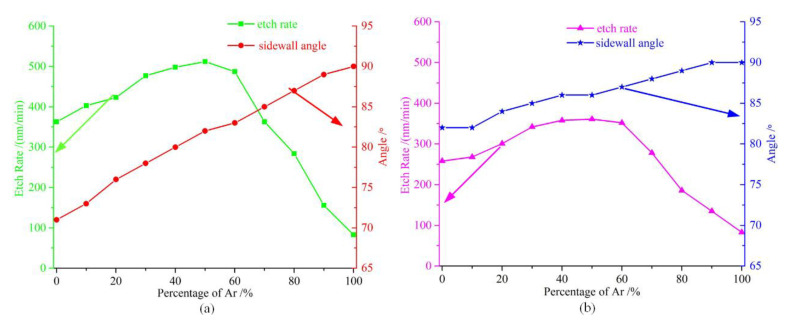
(**a**) The test results of the etching rate and sidewall angle versus the C_4_F_8_ percentage; (**b**) the test results of the etching rate and sidewall angle versus SF_6_ percentage.

Figure 1a shows the dependence of the quartz etching rate and sidewall angle with a Cr mask versus the C_4_F_8_ percentage in the mixture with Ar. The C_4_F_8_ flow ratio ranged from 0% to 100%. It can be seen that the reduction in the C_4_F_8_ percentage resulted in an improvement in the sidewall angle. However, when the C_4_F_8_ flow ratio was less than 50%, the etching rate increased with the C_4_F_8_ percentage increasing due to the insufficiency of the radicals. Moreover, with a high percentage of C_4_F_8_ in the total flow rate, most of the reactive gases were radicals, which resulted in the etching rate keeping at a high level. Furtermore, the increasing C_4_F_8_ flow ratio resulted in an increase in the passivating neutral-to-ion flux ratio leading to a more tapered profile due to the increasing re-deposition of C-rich by-products on the sidewalls, as explained by Bliznetsov in 2015 [22]. To ensure that the gas mixture has sufficient reactive etchants and to keep the etched sidewall profile at an approximate vertical position, it is better to keep the C_4_F_8_ flow ratio within the range of 40~70%.

In order to avoid influence by the C-rich by-products, SF_6_ was introduced along with Ar to determine its effect on the deep quartz etching. As Figure 1b shows, it can be observed that the mixture of SF_6_ and Ar led to an improvement of the sidewall angle, because of the disappearing of the re-deposition by-products. Furthermore, the increasing of the SF_6_ flow rate resulted in a slower reduction in the sidewall angle than that of C_4_F_8_. However, it was evident that the etching rate was significantly lower in contrast to C_4_F_8_, probably because the ion energies and numbers of SF_6_ were less than that of C_4_F_8_ at same flow rate. Thus, it is conducive to the improvement of the sidewall profile by adding SF_6_ into the gas chemistry in the deep quartz etching process. Furthermore, the increasing SF_6_ levels lead to a deterioration of the sidewall angle, which probably results from the increasing of sulfur content.

#### 3.1.2. Mixture of C_4_F_8_/ SF_6_/Ar 

**Figure 2:**
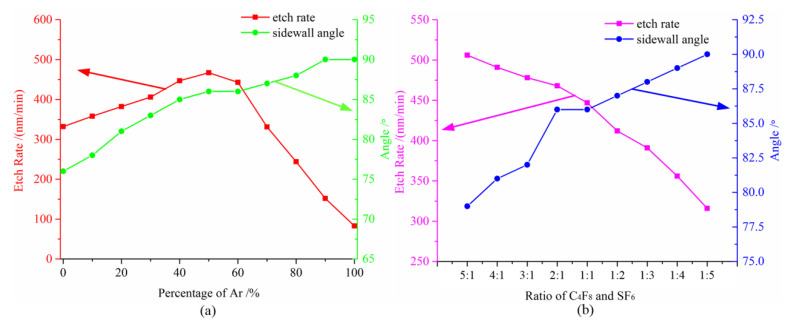
(**a**) The test results of the etching rate and sidewall angle versus the Ar percentage; (**b**) the test results of the etching rate and sidewall angle versus the ratio of C_4_F_8_ and SF_6_.

From the above experiments it is clear that the combination of C_4_F_8_/Ar or SF_6_/Ar is not the optimal decision to achieve deep quartz etching. Thus, a combination of C_4_F_8_, SF_6_ and Ar was selected as the etching media. Figure 2a shows the etching rates of the quartz as a function of the percentage of the Ar flow rate to the total flow rate (Ar + reactive gases). It is obvious that the etching rate is improved. In addition, the Ar flow ratio was kept at 50%, a nearly vertical (87°) sidewall profile was produced. Compared to C_4_F_8_/Ar, it can be seen that the sidewall profile is improved with no deterioration in the etching rate. Compared to SF_6_/Ar, it is obvious that the etching rate is improved significantly. The sidewall profile is acceptable when the percentage of Ar is higher than 50%. In other words, the three-gas mixture is conducive to improving the etching process technology.

A further study was implemented by changing the rate of C_4_F_8_ and SF_6_, in order to investigate the effect of the carbon content. Results for the etch rate and sidewall angle versus the rate of C_4_F_8_ and SF_6_ in the mixture for quartz wafers are presented in Figure 2b (the percentage of Ar was kept at 50%). It is seen that when the rate of C_4_F_8_ and SF_6_ changes from 5:1 to 1:5, the etching rate decreases by almost two-fold. In comparison, the verticality of profile increases to 89.8°. This result indicates that the percentage of the C_4_F_8_ flow rate has an optimal range in the total flow rate. In conclusion, to ensure that, the rate of the three chemical gases (C_4_F_8_/SF_6_/Ar) should be approximately maintained at 1:1:2, which can keep the etched sidewall profile at a vertical position. Additionally, an acceptable etching rate also can be obtained in this etching process. Thus, compared to C_4_F_8_/Ar and SF_6_/Ar, the mixture of the C_4_F_8_/SF_6_/Ar process provided both a better etching rate and less by-product re-deposition on the sidewalls. Therefore, we continued the C_4_F_8_/SF_6_/Ar process optimization.

### 3.2. Effect of Bias Power

**Figure 3:**
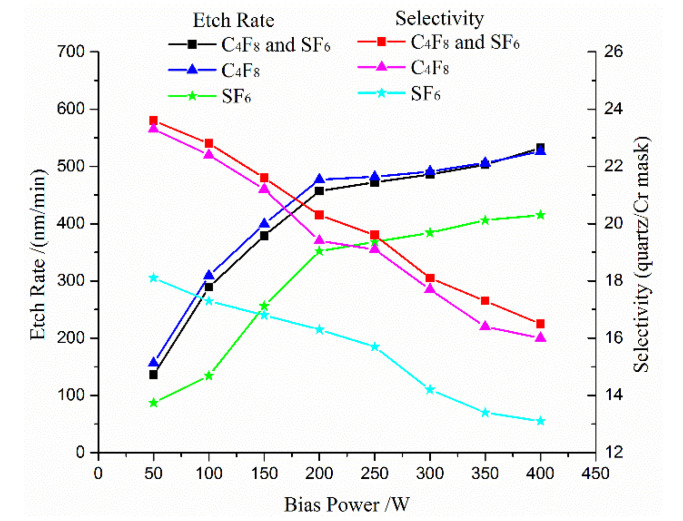
The test results of the etching rate and selectivity influenced by bias power.

The energy of the bombardment component depends on the bias power, which has an effect on the etching rate and selectivity. A test was carried out to investigate the influence on the etching rate and selectivity induced by the bias power. Figure 3 presents the quartz etching rate and selectivity versus bias power. With the decrease in bias power, it is seen that the selectivity drastically improved, and the etch rate dropped. When the bias power changed from 50 W to 400 W, the etch rates increased to 532 nm/min. This result indicates that the bias power has a direct influence on etching efficiency. However, the selectivity for Cr mask declined 46.5% at the same case. 

**Figure 4:**
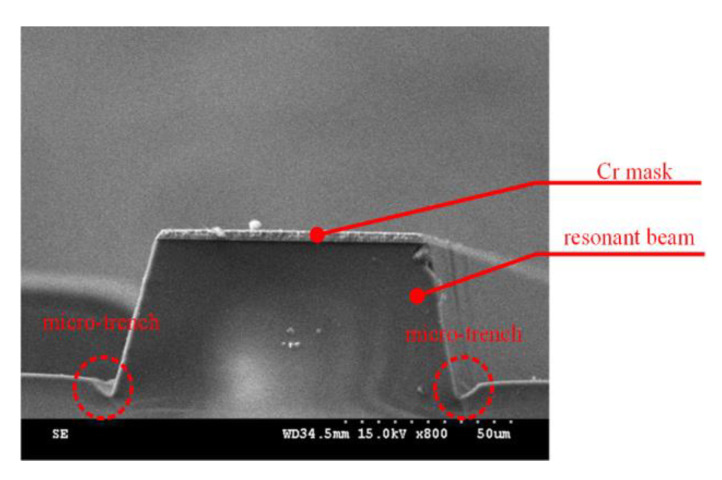
A micro-trench at the position between the sidewall and the bottom surface.

**Figure 5:**
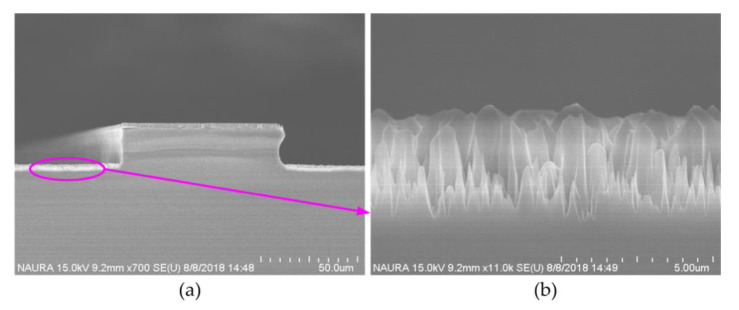
(**a**) Scanning Electron Microscope (SEM) picture of the rough etching bottom; (**b**) SEM picture of the enlarged partial bottom.

In addition, high bias power resulted in a micro-trench at the position between the sidewall and the bottom surface, as shown in Figure 4. Furthermore, when bias power is the excessive high, the etching bottom is rough as shown in Figure 5, which results from the bombardment with much high energy. Thus, to strike a balance between selectivity and etch rate, an appropriate bias power should be chosen for the deep quartz etching process.

### 3.3. Effect of ICP Power 

**Figure 6:**
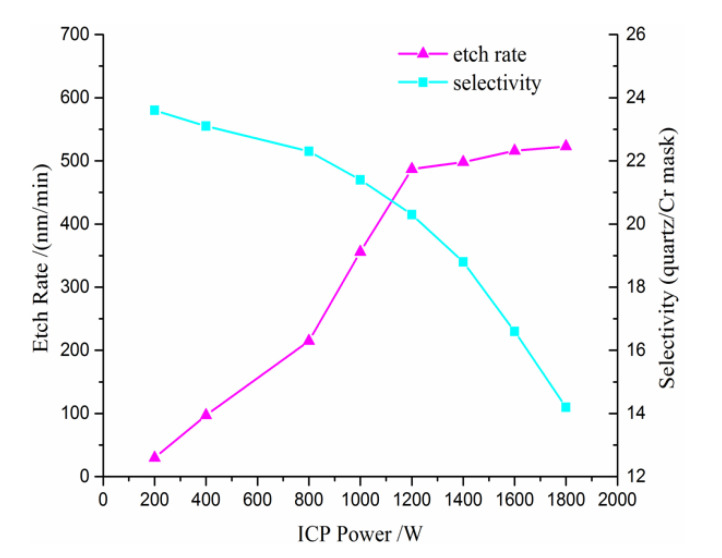
The etch rate of the quartz and selectivity versus the inductively coupled plasma (ICP) power.

The ICP power has an influence on the dissociation rate of the chemical gas. In other words, a higher dissociation rate leads to a higher etching rate. In Figure 6, the etch rate of the quartz and selectivity are plotted versus the ICP power, while keeping bias power = 200 W, gas pressure = 6 mT and gas total flow rate = 110 sccm. It is seen that the etch rate increases with ICP power, due to the increase in the reactive ion flux to the substrate surface. In other words, the increasing ICP power leads to the concentration of the plasma and the dissociation rate of the chemical gas increases correspondingly. Additionally, when the ICP power increases from 1200 W to 1600 W, the increase trend of the etch rate is diminished, which is caused by the fact that the most chemical gas has been ionized when the ICP power reaches 1200 W. However, the selectivity decreases with the ICP power increase. When the ICP power increases from 1200 W to 1600 W, the decrease trend of selectivity is enhanced. Thus, it is obvious that the optimal ICP power is 1200 W.

### 3.4. Effect of Chamber Pressure

**Figure 7:**
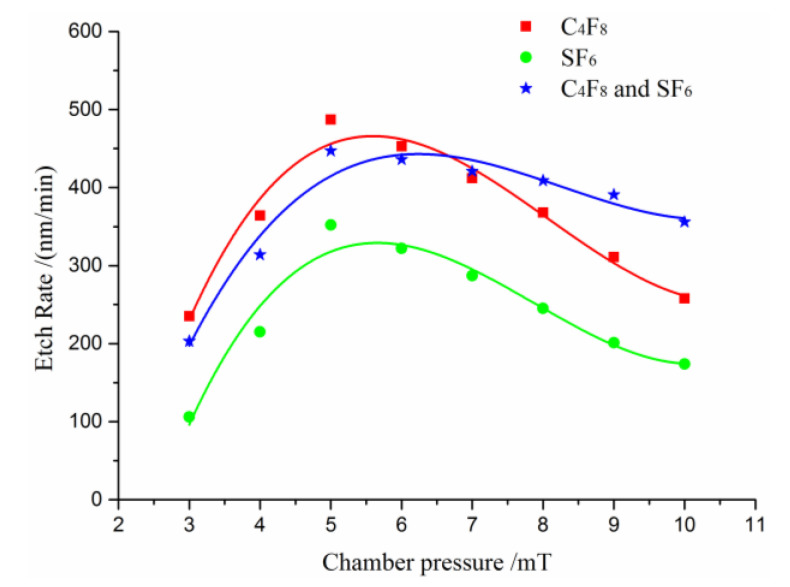
The relationship between the chamber pressure and etch rate.

Experiments were implemented to investigate the relationship between the chamber pressure and etch rate. Figure 7 shows the etch rates of the quartz as a function of the chamber gas pressure. The pressure ranges from 3 mT to 10 mT. When the chamber pressure is lower than 5 mT, it is observed that lower pressure leads to a slower quartz etch rate. This may be due to the fact that at lower pressure the concentrations of radicals are highly reduced and the etching rate is diminished; however, when the chamber pressure is too high, the frequency of ion–ion scattering collisions is highly increased due to the reduction of the mean free path of ions, which results in a decrease in the effects of ion bombardments and a reduction in the quartz etch rate.

Compared with the single gas C_4_F_8_ and SF_6_, the mixture gas of C_4_F_8_ and SF_6_ show a better performance when the chamber pressure is greater than 5 mT. It is observed that the etch rate reduces slowly when pressure increases from 5 mT to 10 mT. It is believed that the presence of fluorine and C_x_F_y_ ions in the plasma, due to high pressure, results in a reduction in concentration of the reactive etchants in this case. Therefore, the chamber gas pressure should be maintained within 4.5~6.5 mT.

### 3.5. Effect of Mask

**Figure 8:**
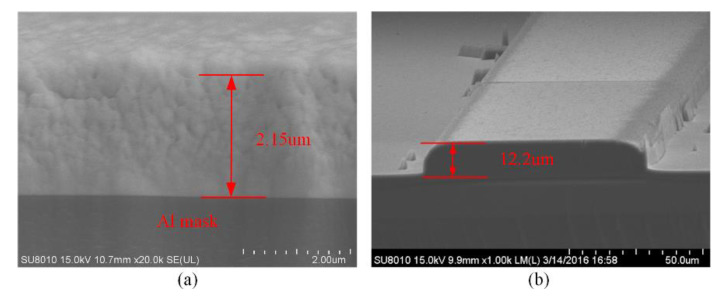
(**a**) SEM image of Al mask before the etching; (**b**) SEM image of the etch profile with Al mask.

**Figure 9:**
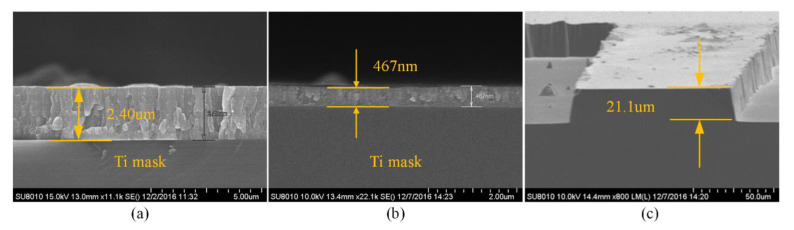
(**a**) SEM image of Ti mask before the etching; (**b**) SEM image of Ti mask after the etching; (**c**) SEM image of the etch profile with Ti mask.

**Figure 10:**
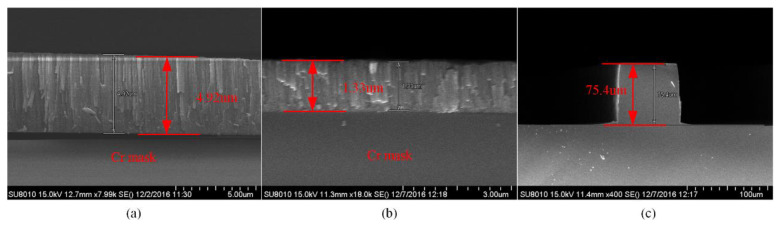
(**a**) SEM image of Cr mask before the etching; (**b**) SEM image of Cr mask after the etching; (**c**) SEM image of the etch profile with Cr mask.

Three kinds of metal masks (Ti, Al and Cr) were investigated to achieve high etch selectivity in this work—note, the ICP power, bias power, etch gas and chamber pressure are the same and shown in Table 1. The etch rate, selectivity and profile were measured for quartz with an original thickness of 300 μm. As shown in Figure 8, for the Al mask with an original thickness of about 1.8 μm, the selectivity is approximately 6 and the profile is less than 76°. For the Ti mask with an original thickness of approximately 2.4 μm, the selectivity is 10.92 and the profile is less than 84°, as shown in Figure 9. For the Cr mask with an original thickness of about 4.92 μm, the selectivity is 20.06 and the profile is vertical, as shown in Figure 10.

Table 1 shows the acquired highest quartz etch rates from all the design of experiment (DOE) runs performed in this test. The highest quartz etch rate was acquired using the C_4_F_8_ and SF_6_ gas combination. According to the research results above, the initial set of etching parameters are listed in Table 1. It can be found that the Cr mask can obtain a higher etch rate and selectivity. The sidewall profile with a Cr mask is more vertical in contrast to that with the Ti mask and Al mask. Moreover, in the sputtering process, the adhesion performance of Cr is superior to the others, which benefits the fabrication of a thicker mask. Hence, the Cr mask is a more appropriate choice for the deep quartz etch process.

### 3.6. Preparation of the Thick Cr Mask

**Figure 11:**
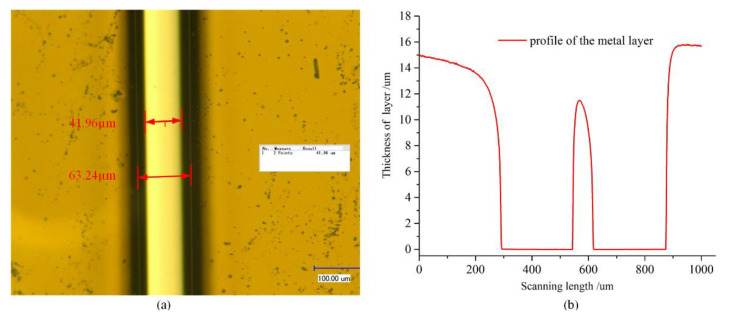
(**a**) The picture of Cr layer under a light microscope; (**b**) the profile of Cr layer with the step profiler.

In order to achieve deep etching, a thick Cr layer should be prepared. Normally, to reduce the production time, the electrodeposited technology is applied to manufacture a thick metal layer. However, it is difficult to pattern the metal layer, and the verticality of the sidewall inclines downward with the increase in thickness, which has an undesirable effect on the sidewall and profile of the etched quartz, as shown in Figure 11. It is obvious that the metal layer fabricated by electrodeposited technology is not suitable for microstructure. Thus, the Magnetron sputtering process is selected to deposit the mask layer.

**Figure 12:**
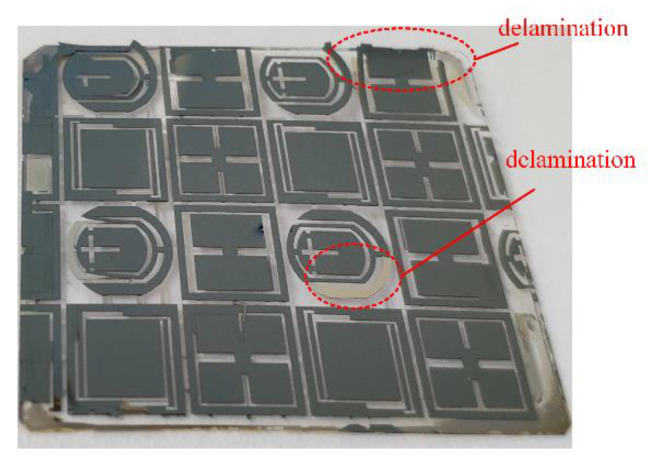
The delamination issue of the Cr layer induced by residual stress.

**Figure 13:**
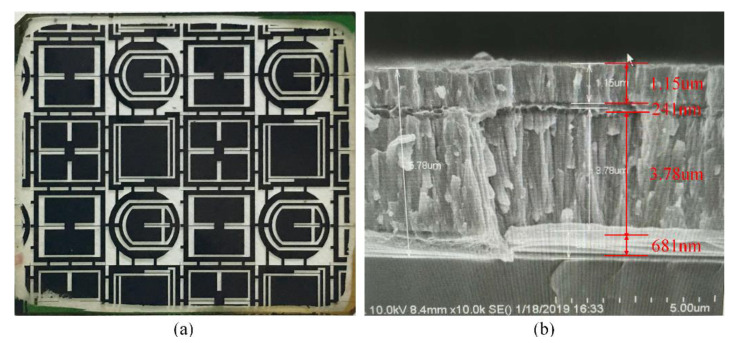
(**a**) The picture of the manufactured mask layer; (**b**) the SEM image of the “sandwich” structure.

It is noticeable that the residual stress increases in the Cr layer with the thickness increase. As shown in Figure 12, the delamination issue of the Cr layer is induced by residual stress when the thickness is increased. In order to overcome the delamination issue, a “sandwich” structure composed of Al layer-Cr layer-Al layer-Cr layer is proposed, as shown in Figure 13. The Al film can play the role of releasing stress and protecting gold electrodes, which can enhance the thickness of the metal mask. In addition, the temperature of the Magnetron sputtering process is less than 50 °C. The ultrasonic cleaning machine is used for achieving a clean lift-off process, and the picture of the manufactured mask layer is shown in Figure 13a.

### 3.7. Statistic of the Experiments

In order to verify the repeatability and feasibility of the quartz etching technology, three repeated experiments were carried out and tested. In each experiment, five samples are selected and tested. The parameters of the optimized etching process are shown in Table 2. The etching time is 155 min and the desired etching depth is 70 μm. The etching depth of each sample was measured, and the statistical results of the experiments are shown in Table 3. It is clear to see that the etching rate of the quartz is about 0.45 μm/min. The statistical results demonstrate that the repeatability and consistency of the process is good.

## 4. Discussion

### 4.1. Summary of the Quartz Deep Etching Technology

**Figure 14:**
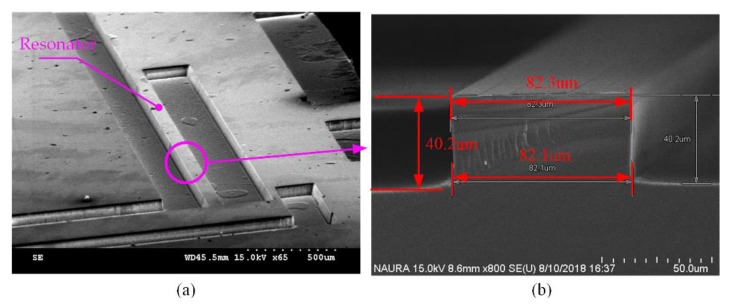
(**a**) SEM image of etched quartz resonator; (**b**) the cross-section view of resonator.

The quartz deep etching process was performed with a deep reactive ion etching (DRIE) system in this paper. The optimal technological results for three kinds of etching gas are shown in Table 4. It can be observed that the gas chemistry of C_4_F_8_/SF_6_/Ar is the optimization. The SEM images of the etch resonator profile and cross-section view are shown in Figure 14. It can be seen that the sidewall is vertical, as shown in Figure 14b. It is feasible to manufacture quartz resonators with proposed dry deep etching process technology.

As shown in Table 1, the etch rate for C_4_F_8_/SF_6_/Ar is significantly lower when using an Al mask and a Ti mask than with a Cr mask. Al, while resistant to RIE thinning, is still sputtered somewhat and redeposits on the target material forming a layer which inhibits etching. This effect is alleviated for increasing Ar proportions. Cr does not noticeably reduce the etch rate because there is no shadow mask. The etching rate is very low in the low bias voltage when SF_6_ is the etch gas, mainly because the effective ions are lesser in contrast to C_4_F_8_. When the bias is increased, the etch rate of the C_4_F_8_ exceeds that of SF_6_, which states that CFx is more suitable as the plasma constituent in etching quartz than SFx. When the C_4_F_8_ proportion is increased overly, the etching rate reduces because of the forming polymer on top of quartz. 

**Figure 15:**
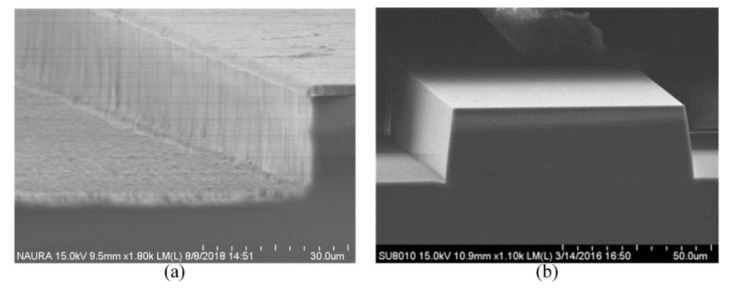
(**a**) The SEM image of the etched surface without clean step; (**b**) the SEM image of the etched surface with clean step.

Only when the bias power is sufficient, does the etching takes place because of two reasons; (1) the steady-state polymer is thinner with higher ion energies and (2) the ion energies are high enough to break the oxide bonds. However, the roughness of the etched surface deteriorates when the bias power is excessively high. In order to reduce the surface roughness, the bias power is investigated to obtain a smooth surface. The lower bias power is beneficial and improves the smoothness and flatness of the etched surface. Moreover, during the etching process, a clean step with Ar and O_2_ is employed to clear away the by-product every 10 min. The parameters of clean step are shown in Table 5. The SEM images of the etched surface are shown in Figure 15. It is obvious that the clean step is conductive to the improvement of the etched surface.

In this work, the focus of our attention is the smoothness of the etched surface and verticality of the sidewall. Moreover, an appropriate etching rate should be obtained to realize a deep etching depth. Table 6 shows the characteristics of the proposed etch technology compared with the previously published literatures. Compared with other research, the results proposed in this work can obtain a higher performance, such as profile and etch rate, although the selectivity is less than that proposed by V. Bliznetsov [22]. However, the mask material presented by V. Bliznetsov was AlN, which is defective as the AlN is unaccommodated to the fabrication of a thick mask. In the sputtering and evaporation process, the deposition rate of AlN is much less than that of Cr (the deposition rate of AlN is about 3 nm/min; the deposition rate of Cr is about 20 nm/min). From the research carried out by K. Kolari [42], it can be seen that the selectivity with a Ni mask is near to a Cr mask; in the research carried out by M. Esashi [9], a higher selectivity was achieved, possibly because the etching depth is shallower than that in this work. Nevertheless, the verticality of the sidewall presented by M. Esashi and K. Kolari is less than that presented in this paper, which has a significant effect on the performance of quartz resonators. Thus, the etch technology presented in this paper is more suitable for dry deep quartz etching.

**Figure 16:**
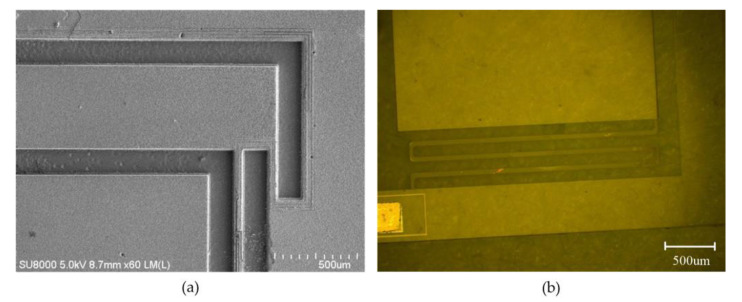
(**a**) SEM picture of the etched quartz structure; (**b**) the picture of the etched quartz structure under a light microscope.

It is worthwhile to mention that the substrate temperature is also a significant factor for reducing the roughness of the sidewall and etching bottom, since a thick polymer layer deposited on the sidewalls and bottom could induce a micro-masking effect, which is a possible factor causing the etched sidewall and bottom roughness. As measured by using the ellipsometer, there were no detectable polymer layers deposited on the samples during the etching. The reason for this was that the substrate temperature was relatively high (90 °C) in our case, so that the deposited polymer layer may been too thin to be detected. This could be of benefit to the reduction in the sidewall and bottom roughness of the etched trench. The pictures of the etched quartz structure with a scanning electron microscope (SEM) and a light microscope are shown in Figure 16. They indicate that the proposed deep quartz etch process technology is credible and feasible.

### 4.2. Compared with Other Quartz Etching Technology

**Figure 17:**
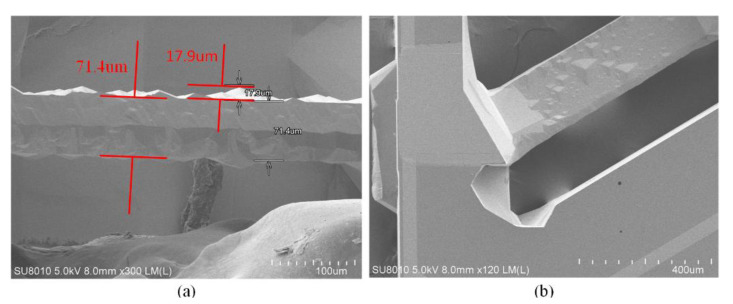
(**a**) The profile of the etched sidewall and bottom with wet chemical etching technology; (**b**) the smoothness of etched bottom wet chemical etching technology.

**Figure 18:**
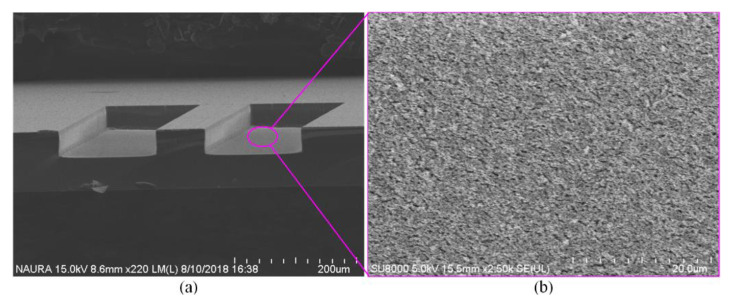
(**a**) The profile of etched sidewall and bottom; (**b**) the roughness of etched bottom.

The structure of the etched quartz with wet chemical etching technology is shown in Figure 17. There are some pyramid structures on the etched sidewall and bottom, which have an adverse effect on the performance of quartz resonators such as stability, reliability and accuracy. It is clear to see that the smoothness of the sidewall and bottom is poor; hence the performance of wet chemical etching technology needs to be improved to meet the requirements of quartz resonators fabrication. The structure of the etched quartz with the proposed deep quartz etch process technology is shown in Figure 18. It is obvious to see that the profile of the etched sidewall and bottom is smooth, which is very necessary and significant to manufacture a quartz resonator.

**Figure 19:**
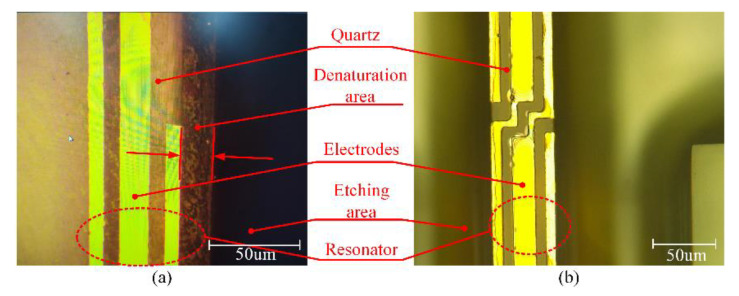
(**a**) The picture of the etched quartz structure with laser ablation under a light microscope; (**b**) the picture of the etched quartz structure with plasma etching under a light microscope.

The ECDM and laser ablation techniques are also used for glass-based material etching in some structures such as micro-channels and holes. The etching rate of them is faster than that of the plasma-based etching technique. However, the ECDM and laser ablation techniques are not suitable for alpha quartz crystal resonant device fabrication. In general, the resonant device structures are much more complex than the micro-channels and holes, and thus the fabrication efficiencies of ECDM techniques and laser ablation are too low to manufacture complex structures and mass production. Moreover, the laser energy results in a quartz crystal denaturation at the edge of the resonant beam in laser ablation technology, as shown in Figure 19a; the roughness and flatness of the etched bottom and sidewall cannot meet the accuracy requirements of the resonator in laser ablation and ECDM technology, which induces an adverse effect on the performance of the quartz resonators. Therefore, the plasma etching technology should be a better choice for quartz resonant device fabrication and the etched quartz resonator is shown in Figure 19b. It is obvious to see that the quartz resonator etched by the proposed etching technology is feasible.

**Figure 20:**
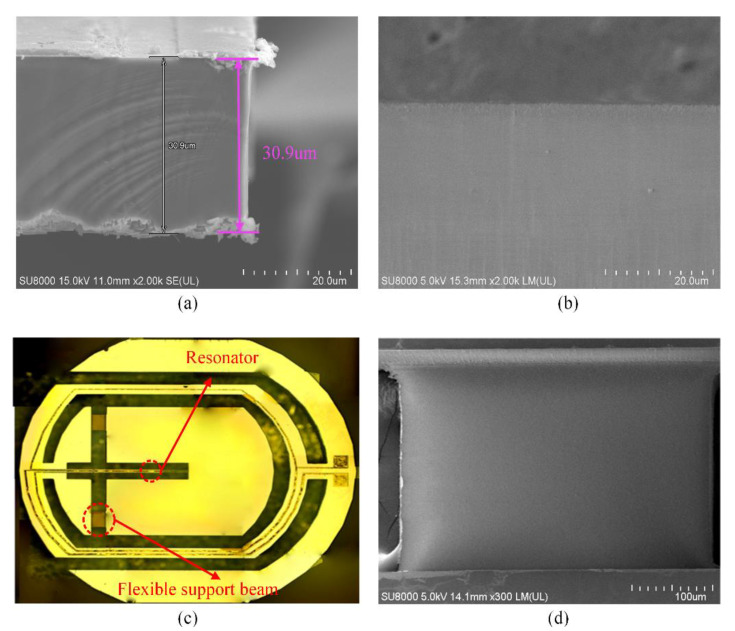
(**a**) The cross-section SEM image of the etched quartz resonator; (**b**) the SEM image of the etched sidewall; (**c**) the picture of the etched MEMS resonant device; (**d**) the SEM image of the etched bottom.

The pictures of the etched MEMS resonant device and the smoothness of the etched surface are shown in Figure 20. The thickness of the quartz resonator is about 30.9 µm, which is very close to the desired thickness (30 µm). The etched sidewall of the resonator is shown in Figure 20b; it is obvious to see that the etched sidewall is smooth and complanate. The etched bottom of the flexible support beam is shown in Figure 20d, which is smooth and complanate. Therefore, the proposed quartz etching technology can obtain a smooth and complanate etched surface, and the accuracy of the etching technology can meet the requirements of MEMS quartz resonant devices.

## 5. Conclusions

In this paper, we investigated the optimization of deep etching with hard masks for quartz crystal. Comparing C_4_F_8_/Ar, SF_6_/Ar and C_4_F_8_/SF_6_/Ar, the third one provided the fewest by-products re-deposited on the sidewalls and bottom, enabled an etching sidewall closer to vertical and an acceptable etching rate. Hence, it is the most suitable for deep quartz etching and high aspect ratio etching. Research on bias power and ICP power were carried out to optimize the etching process parameters. Out of the three studied metal masks, Al, Ti and Cr, the last mask yields the highest selectivity and etching rate. Moreover, the profile of the sidewall with a Cr mask is nearly vertical due to the reduction in by-products re-deposited on the sidewalls and bottom. An optimized etching process with C_4_F_8_/SF_6_/Ar generated an etched trench with a depth of 75.4 µm. The smoothness of the etched surface and verticality of the sidewall meet the requirements of MEMS resonant device fabrication. Based on the confirmed selectivity and Cr mask, it can be expected that the etching capability presented in this work is extendable to the etching of quartz to a depth of around 100 µm.

## Figures and Tables

**Table 1:** Etch process for each metal mask.

Parameter	Al Mask	Ti Mask	Cr Mask
ICP power (W)	1200	1200	1200
Bias power (W)	200	200	200
Chamber pressure (mT)	5	5	5
C_4_F_8_ (sccm)	30	30	30
SF_6_ (sccm)	30	30	30
Ar (sccm)	60	60	60
Etch rate of quartz (nm min^−1^)	0.2	0.23	0.42
Selectivity (quartz/mask)	6	10.92	21.06
Profile (°)	76	84	89

**Table 2:** The parameters of optimized etching process.

Parameters	ICP Power (W)	Bias Power (W)	Chamber Pressure (mT)	Temperature (°C)	Ar (sccm)	C_4_F_8_ (sccm)	SF_6_ (sccm)	Etching Time (min)
Value	1200	230	6	90	60	30	30	155

**Table 3:** Statistical results of three repeated experiments.

	Experiment	Run 1	Run 2	Run 3
Sample				
1		70.1 μm	69.7 μm	70.1 μm
2		70.4 μm	69.3 μm	70.2 μm
3		70.3 μm	69.1 μm	69.8 μm
4		69.9 μm	70 μm	69.6 μm
5		70.5 μm	69.5 μm	70 μm
Average value		74.24 μm	69.52 μm	69.94 μm
Standard deviation		0.24083	0.34928	0.24083
Etching rate (μm/min)		0.453	0.449	0.451

**Table 4:** The results of quartz etching with three chemical gases.

Etch Gas	Etch Rate of Quartz (μm/min)	Profile (°)
C_4_F_8_/Ar	0.48	82
SF_6_/Ar	0.31	86
C_4_F_8_/SF_6_/Ar	0.43	90

**Table 5:** The parameters of clean step.

Parameters	ICP Power (W)	Bias Power (W)	Chamber Pressure (mT)	Temperature (°C)	Ar (sccm)	O_2_ (sccm)
Value	1200	200	5	90	40	15

**Table 6:** The characteristics of the proposed etch technology compared with the previously published literatures.

Parameters	Target	Mask	Etch Rate (μm/min)	Selectivity	Profile (Degree)	Depth (μm)
H. Chen [37]	quartz	SU-8	0.22	0.34	86	55
V. Bliznetsov [22]	USG	AlN	0.32	49	88	21
Tathagata Ray [41]	Fused silica	KMPR^®^1025	0.0933	1.13	85	30
M. Esashi [4]	quartz	Ni	0.5	30	87	20
K. Kolari [38]	PyrexTM	Ni	0.7	23.3	78	100
This work	quartz	Cr	0.45	21.06	90	75.4
